# Preclinical efficacy of drug delivery systems in colon cancer therapy: a systematic review and meta-analysis of in vivo animal studies

**DOI:** 10.1186/s43046-026-00365-8

**Published:** 2026-05-19

**Authors:** Rozafa Koliqi, Arlinda Daka Grapci, Michael Y. Henein, Agata Bielecka-Dabrowa, Ibadete Bytyçi

**Affiliations:** 1 Medical Faculty, University of Prishtina, Prishtine, Republic of Kosovo; 2 Imperial College, London, UK; 3 Medical University of Lodz, Lodz, Poland

**Keywords:** Colon cancer, Drug delivery systems, Nanoparticles, Liposomes, Micelles, Preclinical, Chemotherapy, Targeted therapy, Tumor growth inhibition

## Abstract

**Background:**

Drug delivery systems (DDS) offer a promising strategy to enhance the therapeutic index of chemotherapeutic agents in colon cancer by improving tumor targeting, circulation time, and controlled drug release. Despite extensive preclinical research, a quantitative synthesis evaluating the efficacy of DDS and the influence of design parameters remains lacking.

**Methods:**

We conducted a systematic review and meta-analysis of preclinical in vivo studies assessing DDS-based chemotherapy in murine models of colon cancer. A comprehensive search of PubMed, EMBASE, Scopus, Google Scholar, Cochrane CENTRAL, and ClinicalTrials.gov was performed through October 15, 2025. Outcomes included tumor growth inhibition, with subgroup analyses examining the effects of DDS platform, chemotherapeutic agent, targeting strategy, ligand type, and route of administration.

**Results:**

Twenty-three studies comprising 25 experiments and 539 animals were included. Overall, DDS-based therapies significantly reduced tumor growth compared with controls ((WMD: − 557.7; 95% CI: − 716.8 to − 398.5; I²=88%; *p* < 0.001) and free-drug administration ((WMD: − 276.3; 95% CI: − 367.6 to − 185.1; I²=78%; *p* < 0.001). Both targeted and non-targeted DDS significantly reduced tumor growth compared with free drug treatment, while targeted DDS showed significantly greater efficacy than non-targeted systems (WMD: − 240.3; 95% CI: − 399.4 to − 81.25; I²=59%; *p* = 0.003). Although no statistically significant differences were observed between DDS platforms or chemotherapeutic agent subgroups, micelle-based systems and DDS formulations incorporating SN-38 or doxorubicin tended to show greater tumor growth reduction. Intravenous administration demonstrated significantly greater efficacy than intraperitoneal delivery, while hyaluronic acid- and aptamer-based targeting strategies achieved the largest tumor growth inhibition. Risk-of-bias assessment indicated moderate methodological quality, with variability in reporting of randomization, blinding, and sample size calculations.

**Conclusions:**

DDS-based chemotherapy consistently improves antitumor efficacy in preclinical colon cancer models, with targeting strategy, platform type, chemotherapeutic agent, and administration route influencing outcomes. These findings support the rational design of DDS platforms and underscore their translational potential. Rigorous preclinical study design and standardized reporting of efficacy and safety are essential to facilitate clinical translation.

**Supplementary Information:**

The online version contains supplementary material available at 10.1186/s43046-026-00365-8.

## Introduction

Colon cancer remains one of the leading causes of cancer-related morbidity and mortality worldwide. Despite advances in early detection and multimodal therapy, the global burden of colon cancer continues to increase, particularly in both aging populations and younger individuals exposed to lifestyle-related risk factors such as obesity, sedentary behavior, and dietary patterns. Metastatic disease develops in approximately half of patients and represents the principal determinant of poor prognosis and cancer-related mortality [1–3]. Consequently, optimizing therapeutic strategies aimed at improving tumor control while minimizing systemic toxicity remains a major clinical priority.

Current management of colon cancer relies on a combination of surgery and systemic chemotherapy. Standard therapeutic approaches typically involve neoadjuvant chemotherapy followed by surgical resection and adjuvant chemotherapy, or primary surgical treatment followed by systemic therapy depending on disease stage and patient characteristics [4]. Although conventional intravenous (IV) chemotherapy remains a cornerstone of treatment, its clinical utility is frequently limited by suboptimal pharmacokinetics, short circulation half-life, non-specific biodistribution, and significant systemic adverse effects including myelosuppression, neurotoxicity, and gastrointestinal toxicity [5, 6]. In patients with peritoneal dissemination, combined intraperitoneal (IP) and IV chemotherapy may enhance local drug exposure; however, treatment-related toxicity and limited targeting precision remain substantial challenges [7]. These limitations highlight the urgent need for innovative delivery strategies that improve therapeutic selectivity and enhance antitumor efficacy.

Drug delivery systems (DDS) have emerged as promising technological platforms designed to optimize the therapeutic index of anticancer agents. Nanoparticles, liposomes, polymeric micelles, and other engineered carrier systems have been developed to enhance drug solubility, improve circulation time, and enable controlled or sustained drug release. By facilitating preferential drug accumulation within tumor tissues, DDS may increase local therapeutic concentration while reducing systemic exposure and toxicity [8]. Targeting strategies employed in DDS include passive targeting mechanisms, such as the enhanced permeability and retention effect, as well as active targeting approaches involving the conjugation of ligands, antibodies, aptamers, or other molecular moieties that enable selective interaction with tumor-specific receptors [9]. These advances have significantly expanded the potential of DDS to overcome pharmacological barriers associated with conventional chemotherapy.

The therapeutic performance of DDS is influenced by multiple design and biological factors, including carrier composition, physicochemical characteristics, particle size, surface modification, and route of administration. Differences between IV and IP delivery routes may substantially affect drug biodistribution, tumor penetration, and systemic exposure [10, 11]. Additionally, materials used in DDS construction, including polymers, lipids, proteins, and inorganic components, play an important role in determining drug release kinetics, stability, and biological interactions [12]. Although numerous chemotherapeutic agents have been encapsulated within DDS platforms for colon cancer therapy, the optimal combination of delivery platform, targeting strategy, and therapeutic payload remains uncertain.

Preclinical research has extensively evaluated DDS technologies in vitro and in vivo, with animal models serving as a critical step toward clinical translation. While many DDS demonstrate promising antitumor activity in experimental colon cancer models, reported outcomes vary widely due to differences in experimental design, tumor models, treatment regimens, and evaluation endpoints. Moreover, DDS-based strategies have shown potential to enhance chemotherapy outcomes across various malignancies, including breast, lung, ovarian, brain cancers, and melanoma, further supporting their broad therapeutic applicability [13–16]. However, despite the growing body of experimental research, findings remain fragmented and lack quantitative synthesis, making it difficult to determine which DDS design features confer the greatest therapeutic advantage.

Importantly, no comprehensive systematic review and meta-analysis has quantitatively evaluated the efficacy of DDS-based chemotherapy specifically in preclinical colon cancer models while simultaneously examining how DDS characteristics such as delivery platform type, targeting strategy, ligand composition, chemotherapeutic agent selection, and route of administration, affect therapeutic outcomes. A rigorous synthesis of preclinical evidence is essential to identify promising DDS designs, guide translational research, and inform the development of future clinical trials aimed at improving chemotherapy efficacy and safety [17, 18].

Therefore, the aim of this systematic review and meta-analysis was to evaluate and compare the therapeutic efficacy of drug delivery systems in preclinical animal models of colon cancer and to assess how specific DDS characteristics, including delivery platform, chemotherapeutic agent, targeting strategy, ligand type, and route of administration, influence tumor growth inhibition. By providing a structured quantitative synthesis of existing preclinical evidence, this study seeks to support the rational development of optimized DDS platforms and facilitate their translation into clinically relevant cancer therapies.

## Methods

### Study design and reporting framework

This study was conducted as a systematic review and meta-analysis in accordance with the Preferred Reporting Items for Systematic Reviews and Meta-Analyses (PRISMA 2020) guidelines [19]. Because this investigation synthesized previously published preclinical animal studies, institutional review board approval and informed consent were not required. The research question, eligibility criteria, and outcomes were predefined using the PECOS framework (Population, Exposure/Intervention, Comparison, Outcomes, Study design), as summarized in *Table S1.*

### Search strategy

A comprehensive literature search was performed from database inception through October 15, 2025. The following electronic databases were systematically searched: PubMed/MEDLINE, EMBASE, Scopus, Google Scholar, Cochrane Central Register of Controlled Trials (CENTRAL), and ClinicalTrials.gov. The search strategy combined controlled vocabulary and free-text terms related to colon cancer, drug delivery systems, chemotherapy, and animal models. Core search terms included: (“colon cancer” OR “colorectal cancer”) AND (“drug delivery system*” OR DDS OR nanoparticle* OR liposome* OR micelle*) AND (“chemotherapy” OR chemotherapeutic* OR “targeted therapy”) AND (“animal model*” OR mice OR murine) AND (“tumor growth” OR therapeutic efficacy OR treatment response). Detailed search strategies are provided in *Table S2.*

To ensure comprehensive coverage, reference lists of relevant reviews and eligible studies were manually screened. Abstracts and proceedings from major oncology conferences, including the American Association for Cancer Research (AACR), European Society for Medical Oncology (ESMO), American Cancer Society (ACS), and Cancer Research UK meetings, were also examined. Searches were restricted to English-language publications, and no publication date limits were applied.

### Study selection

Two independent reviewers (R.K. and I.B.) screened titles and abstracts for eligibility. Full-text articles of potentially relevant studies were subsequently assessed independently. Discrepancies were resolved through discussion and consensus.

Studies were included if they:

- investigated drug delivery systems delivering chemotherapeutic agents in in vivo murine models of colon cancer;
- reported tumor growth inhibition or quantitative tumor response outcomes;
- provided information on at least one DDS characteristic (e.g., drug payload, ligand type, route of administration, or targeting strategy);
- included a minimum follow-up period of one month.

Studies were excluded if they:

- lacked sufficient quantitative outcome data;
- used non-murine animal models;
- investigated cancer types other than colon cancer;
- were ongoing studies without relevant interim data.

### Data extraction and risk of bias assessment

Data extraction was independently performed by the same two reviewers (R.K. and I.B.) using a standardized data collection form. Extracted variables included study characteristics, animal model details, DDS platform type, chemotherapeutic agent, targeting strategy, ligand characteristics, route of administration, duration of follow-up, and tumor growth outcomes. All extracted data were verified against the original publications to ensure accuracy and consistency.

The methodological quality and risk of bias of included studies were independently assessed using SYRCLE’s Risk of Bias tool for animal studies [20]. Each domain was categorized as low, high, or unclear risk of bias. Disagreements were resolved through consensus.

### Outcome measures

The primary outcome was the therapeutic efficacy of drug delivery systems in preclinical colon cancer models, measured as tumor growth inhibition. Secondary analyses evaluated the impact of DDS design characteristics on treatment outcomes, including:

- type of chemotherapeutic agent (conventional drugs, biologics, or combination therapies);
- ligand characteristics (e.g., peptides, antibodies, or other targeting moieties);
- route of administration (e.g., intravenous vs. intraperitoneal);
- targeting strategy (active targeting versus passive targeting such as enhanced permeability and retention).

### Data synthesis and statistical analysis

Meta-analyses were conducted using Review Manager (RevMan Version 5.1, The Cochrane Collaboration, Copenhagen, Denmark). Summary effects were calculated as weighted mean differences (WMDs) with 95% confidence intervals (CIs). When necessary, mean and standard deviation values were estimated according to the method described by Hozo et al. [21].

A random-effects model was applied to account for between-study variability. Statistical heterogeneity was assessed using the Cochrane Q test and the I² statistic, with I² values interpreted as low (< 25%), moderate (25–50%), or high (> 50%) heterogeneity. Residual heterogeneity was incorporated using tau² estimation via the restricted maximum likelihood method [22].

Subgroup analyses were conducted according to ligand type, route of administration, and chemotherapeutic agent class. Publication bias was evaluated through visual inspection of funnel plots asymmetry and Egger’s regression test. A two-tailed *p*-value < 0.05 was considered statistically significant.

## Results

### Study selection and characteristics

The systematic search identified 2,708 records. After removal of duplicates and initial screening, 142 articles were considered potentially relevant and underwent full-text assessment. Ultimately, 23 studies comprising 25 independent experiments met the predefined inclusion criteria and were included in the quantitative synthesis [23–45]. The study selection process is illustrated Figure S1, and detailed characteristics of included studies are presented in Table S3.

A range of DDS platforms was evaluated across the included experiments. Nanoparticle-based DDS were used in nine experiments [26–30, 34, 35, 39, 40], micelle-based systems in eight experiments [23, 32, 36, 38, 41, 42, 45], and liposomal formulations in six experiments [24, 25, 31, 33, 44]. Materials and physicochemical properties varied considerably among platforms. Active targeting strategies were employed in approximately 45% of experiments, primarily using hyaluronic acid [27, 28], aptamers [36, 43], or folic acid [24, 25, 29, 33, 44] as targeting ligands.

Fluorouracil was the most frequently used chemotherapeutic agent (16 experiments) [24–31, 33–35, 37, 39, 40, 45], followed by doxorubicin [23, 44] and irinotecan/SN-38 formulations [38, 41, 42]. Intravenous administration was the predominant delivery route (73% of experiments) [23, 27, 31, 34–42, 44, 45], whereas intraperitoneal delivery was used in 26% [24–26, 29, 30, 33]. All studies employed murine models; sex distribution included female (38%), male (23%), or unspecified animals (39%). Mouse body weight ranged from 12 to 30 g but was reported in only 30% of studies [29, 30, 34, 37, 40, 41, 43]. Xenograft models predominantly used CT-26, HT-29, and SW-480 cell lines (38%, 23%, and 11.5%, respectively), with the most common inoculation dose being 1 × 10⁶ cells [23–29, 33, 35, 39, 42].

### Overall efficacy of drug delivery systems

At a median follow-up period of 3–6 weeks, DDS-based therapy demonstrated significantly greater tumor growth inhibition compared with control treatment (WMD: − 557.7; 95% CI: − 716.8 to − 398.5; I^2^ = 88; *p* < 0.001). Similarly, DDS-encapsulated chemotherapy showed significantly enhanced antitumor efficacy compared with free drug administration (WMD: − 276.3; 95% CI: − 367.6 to − 185.1; I^2^ = 78; *p* < 0.001; Figure S2).

### Targeted versus non-targeted DDS

**Fig. 1:**
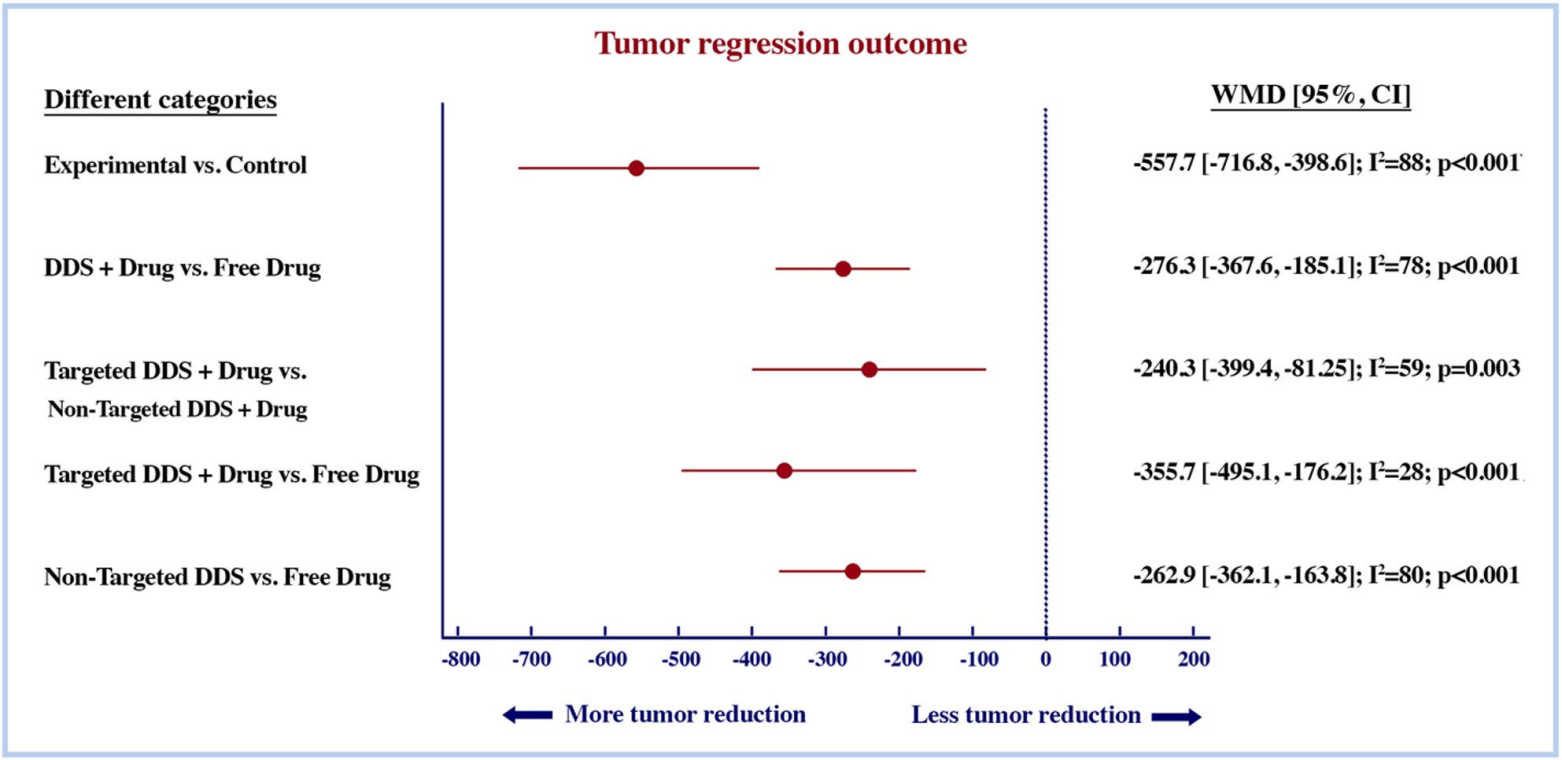
Subgroup analyses of tumor growth inhibition comparing chemotherapeutics delivered via targeted and/or non targeted drug delivery systems (DDS) versus free chemotherapeutics

Subgroup analysis revealed that both targeted and non-targeted DDS combined with chemotherapeutic agents significantly reduced tumor growth compared with free drug alone (WMD: − 335.7; I^2^ = 28 and − 262.9; I^2^ = 80, respectively; *p* < 0.001 for both comparisons). Targeted DDS demonstrated significantly greater tumor growth inhibition compared with non-targeted systems (WMD: − 240.3; 95% CI: − 399.4 to − 81.25; I^2^ = 59; *p* = 0.003; Figure S3). A summary of overall DDS efficacy is shown in Fig. 1.

### Comparison of DDS type

Despite no statistically significant differences being observed between DDS subgroups (Chi² = 1.38; I²=0%; *p* = 0.50), comparative analysis across DDS platforms showed the greatest mean tumor growth reduction with micelle-based systems (WMD: − 365.5; I²=87%; *p* = 0.001), followed by nanoparticle formulations (WMD: − 250.1; I²=79%; *p* = 0.002) and liposomal systems (WMD: − 215.1; I²=0%; *p* = 0.0006), suggesting broadly comparable efficacy among the evaluated DDS platforms (Figure S4).

### Subgroup analyses by chemotherapeutic agent

**Fig. 2:**
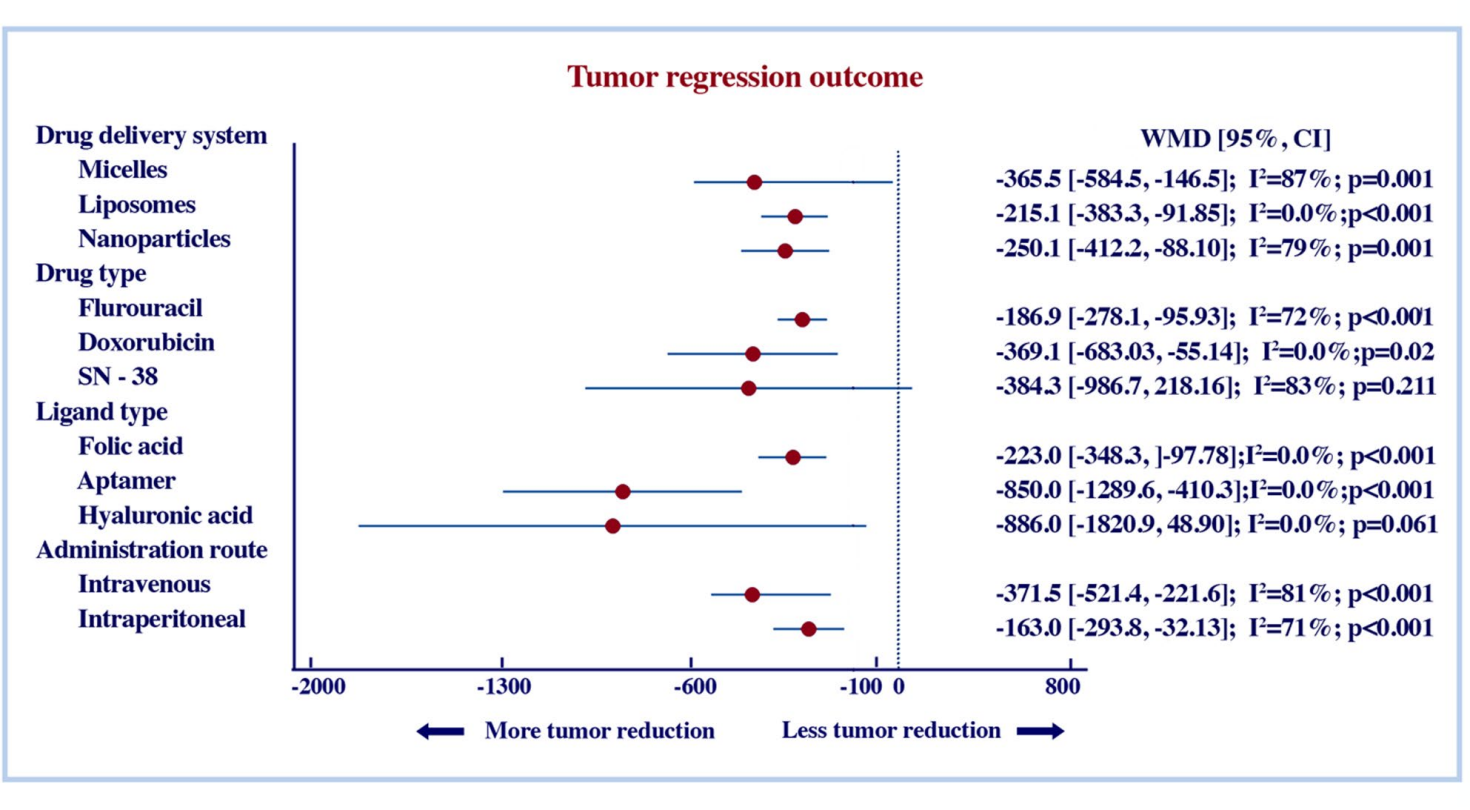
Subgroup analyses of tumor growth inhibition by type types of DDS, chemotherapeutics, ligand and route of administration

DDS formulations incorporating SN-38 tended to show greater reduction in tumor growth (WMD: − 384.3; I²=80%; *p* = 0.21), followed by doxorubicin (WMD: − 369.1; I²=0%; *p* = 0.02), whereas fluorouracil-based DDS exhibited comparatively smaller effects (WMD: − 186.9; I²=72%; *p* < 0.001; Figure S5). Although these differences did not reach statistical significance, the observed trend suggests potentially greater efficacy of SN-38 and doxorubicin when delivered via DDS in experimental colon cancer models (Fig. 2).

### Route of administration and ligand characteristics

Intravenous administration was associated with significantly greater tumor growth inhibition compared with intraperitoneal delivery (WMD: − 371.5 vs. − 163.0; Test for subgroup differences; Chi^2^=4.22; I^2^ = 76%; *p* = 0.04; Figure S6). Analysis of ligand-based targeting strategies indicated that hyaluronic acid-modified DDS achieved the largest reduction in tumor growth (WMD: − 886), followed by aptamer-based systems (WMD: − 850) and folic acid-conjugated DDS (WMD: − 223; *p* = 0.01; Test for subgroup differences; Chi^2^=8.87; I^2^ = 77%; *p* = 0.01; Figure S7). A summary of overall DDS efficacy is shown in Fig. 2.

### Risk of bias assessment

Assessment using SYRCLE’s risk of bias tool indicated moderate overall methodological quality among the included studies (Table S4). Although most studies clearly defined their objectives and primary outcomes, variability was observed in the reporting of randomization methods, blinding procedures, and sample size calculations, representing potential sources of bias. These limitations warrant cautious interpretation of the findings. Future preclinical studies should implement more rigorous study designs and standardized reporting practices to improve reliability and reproducibility.

## Discussion

This systematic review and meta-analysis critically evaluated the therapeutic efficacy of chemotherapeutic agents delivered through drug delivery systems (DDS) in preclinical models of colon cancer, with tumor growth inhibition as the primary outcome. A total of 23 studies comprising 25 independent experiments and 539 animals were included. Overall, the findings demonstrate a consistent and significant enhancement in antitumor activity when chemotherapeutic agents were administered via DDS compared with conventional free-drug formulations, supporting the growing role of nanotechnology-based delivery strategies in experimental oncology.

A central finding of this analysis was the superior efficacy of DDS-encapsulated chemotherapy across all included studies. Improved outcomes are likely attributable to enhanced pharmacokinetic properties, prolonged circulation time, targeted tumor accumulation, and controlled drug release profiles associated with DDS platforms. These characteristics enable increased local drug concentration within tumor tissue while reducing systemic exposure, thereby potentially improving therapeutic index. Such advantages are particularly relevant in colon cancer, where conventional chemotherapeutics are frequently limited by poor solubility, rapid clearance, systemic toxicity, and non-specific biodistribution [46, 47]. The consistent tumor inhibition observed across studies reinforces the potential of DDS strategies to overcome major pharmacological limitations of standard chemotherapy. Subgroup analyses highlighted several design features potentially influencing DDS performance. Both targeted and non-targeted DDS significantly improved tumor control compared with free drug treatment, while actively targeted systems demonstrated numerically greater efficacy. Ligand-mediated targeting strategies, including hyaluronic acid, aptamers, and folic acid, were associated with enhanced tumor inhibition, with hyaluronic acid–modified DDS showing the largest effect. These findings support the potential importance of active targeting approaches in improving DDS functionality and therapeutic precision [29, 31–33]. In addition, intravenous administration was associated with significantly greater tumor growth inhibition compared with intraperitoneal delivery, suggesting that delivery route may influence therapeutic efficacy, potentially through improved systemic distribution and tumor accumulation. Comparative evaluation of DDS platforms showed that micelle-based systems achieved the greatest mean reduction in tumor growth, followed by nanoparticle and liposomal formulations. However, no statistically significant differences were observed between DDS subgroups, suggesting broadly comparable efficacy among the evaluated platforms when appropriately engineered. Similarly, although subgroup differences according to chemotherapeutic agent were not statistically significant, DDS formulations containing SN-38 tended to show greater tumor growth reduction, followed by doxorubicin, whereas fluorouracil-based systems demonstrated comparatively smaller effects. These trends may reflect differences in pharmacodynamic properties and drug delivery characteristics. In particular, DDS platforms may offer advantages for compounds such as SN-38 by improving solubility, stability, and tumor accumulation [9, 48].

Additionally, the route of administration emerged as a significant determinant of efficacy, with intravenous delivery associated with greater tumor inhibition than intraperitoneal administration. This may be related to improved systemic distribution and enhanced tumor targeting via mechanisms such as the enhanced permeability and retention (EPR) effect, underscoring the importance of delivery strategy optimization in translational research.

### Strengths and limitations

This meta-analysis provides a comprehensive quantitative synthesis of preclinical evidence supporting the therapeutic advantages of DDS-based chemotherapy in colon cancer models. The consistent superiority of DDS formulations across included studies supports the continued development of advanced delivery platforms and their potential clinical translation. In addition, subgroup analyses examining targeting strategies, DDS platforms, chemotherapeutic agents, and routes of administration provided further insight into factors potentially influencing therapeutic efficacy. However, these subgroup findings should be interpreted cautiously, as no statistically significant differences were observed between groups.

Several limitations should be acknowledged. Significant heterogeneity was observed across analyses, likely reflecting differences in experimental design, animal models, dosing regimens, and evaluation timelines. In addition, important DDS physicochemical parameters, including particle size, zeta potential, drug loading, and release kinetics, were inconsistently reported, limiting mechanistic interpretation and pooled quantitative analysis. These findings highlight the need for standardized DDS characterization in future studies. Finally, toxicity and safety outcomes could not be comprehensively evaluated because of inconsistent reporting across studies. Methodological assessment using SYRCLE’s risk of bias tool demonstrated moderate study quality, with inconsistent reporting of randomization, blinding, and sample size calculations. Future preclinical studies should adopt more rigorous methodologies, standardized reporting, and comprehensive safety assessments to improve translational relevance.

## Conclusion

This systematic review and meta-analysis demonstrates that DDS improve the antitumor efficacy of chemotherapeutic agents in preclinical colon cancer models. Targeted DDS and intravenous administration were associated with greater tumor inhibition, while micelle-based systems and DDS incorporating SN-38 or doxorubicin tended to show greater efficacy, although subgroup differences were not statistically significant.

Despite methodological heterogeneity and incomplete reporting across studies, DDS consistently demonstrated advantages over free-drug formulations, supporting their potential translational value in colon cancer therapy. Future preclinical studies should adopt more rigorous methodologies, standardized reporting, and comprehensive safety assessments to facilitate clinical translation.

## Supplementary Information

Supplementary Material 1.

## Data Availability

The data supporting the findings of this study are derived from previously published articles cited in the reference list. Extracted data and analysis files are available from the corresponding author upon request.
