# Supplementary material for: Preclinical efficacy of drug delivery systems in colon cancer therapy: a systematic review and meta-analysis of in vivo animal studies
Source: J Egypt Natl Canc Inst. 2026 May 19;38:27. doi: 10.1186/s43046-026-00365-8 (PMC13313309; doi:10.1186/s43046-026-00365-8)
Supplement: Supplementary file 1 — Supplementary Material 1. [file 43046_2026_365_MOESM1_ESM.docx]

**SUPPLEMENTAL MATERIAL**

**­­­­­­­­­**

Efficacy of drug delivery systems in colon cancer: A meta analysis of animal studies

Contents

[**Table S1.** PECOS model 2](#_Toc67823651)

[**Table S2.** The search and screening strategy. 2](#_Toc67823652)

[**Figure S1.** Flow-chart of studies included in the meta-analysis……………………...…………….3](#_Toc67823653)

[**Figure S2.** The efficacy of chemotherapy in inhibiting tumor growth compared to the control group………………………………………...……………………………………………………....4](#_Toc67823653)

[**Table S3**. Main characteristics of papers included in the meta analysis………………………………………………………..…………………………………..5](#_Toc67823653)-7

[**Figure S3**. Comparison of the efficacy of targeted versus non-targeted DDS in combination with drug therapy on tumor growth inhibition. 8](#_Toc67823654)

[**Figure S4**. The efficacy of chemotherapeutics in various DDS types compared to free chemotherapeutics on tumor growth inhibition in subgroup analysis………………………………9](#_Toc67823654)

[**Figure S5**. Tumor growth inhibition effects of different chemotherapeutics in a DDS compared to free chemotherapeutics in subgroup analysis: Fluorouracil, Doxorubicin, SN-38. 10](#_Toc67823654)

[**Figure S6**. The efficacy of chemotherapeutics in DDS compared to free chemotherapeutics on tumor growth inhibition in subgroup analysis: Intravenous, Intraperitoneal 11](#_Toc67823654)

[**Figure S7**. The efficacy of chemotherapeutics in DDS compared to free chemotherapeutics on tumor growth inhibition in subgroup analysis: Intravenous, Intraperitoneal 12](#_Toc67823654)

[**Table S4.** Assessment of risk of bias in the included studies using SYRCLE’s RoB tool for animal studies 13,](#_Toc67823655) 14

**Table S1.** PECOS model

| **PECOS model** | |
| --- | --- |
| **Participants** | **Animal** (**Species:** Mice; **Gender:** Female/Male; **Strains:** CT26, HT29, SW480). |
| **Exposure:** | Studies investigating chemotherapeutics encapsulated in DDS, such as nanoparticles, liposome or micelles, for colon cancer treatment in animal studies. |
| **Comparator/Control** | Applicable |
| **Outcomes** | The efficacy of chemotherapy in DDS compared to free chemotherapy (not in a DDS), on tumor growth inhibition in animal studies. |
| **Study design** | Includes studies evaluating chemotherapeutics encapsulated in DDS for colon cancer treatment in animal studies. Previous systematic reviews were not included; however, these articles were searched and analyzed for individual trials. |

**Table S2.** Literature search strategy

| **Database search** | PubMed and EMBASE |
| --- | --- |
| **Efficacity & safety** | "Colon cancer" OR "Colorectal cancer" AND "Drug delivery systems" OR “DDS” OR "Nanoparticles" OR "Liposomes" OR "Micelles" AND "Chemotherapy" OR "Chemotherapeutics" OR "Targeted therapy" AND "Animal models" OR "Mice" AND "Treatment" OR "Therapeutic efficacy" OR "Tumor growth" |
| **Occurrence term** | "Efficacy" OR "Safety" OR "Tumor growth inhibition" |
| **Additional search** | Relevant scientific organizations and publications, including the American Association |
|  | for Cancer Research (AACR), European Society for Medical Oncology (ESMO), |
|  | American Cancer Society (ACS), and Cancer Research UK. |

**Figure S1.** Flow-chart of studies included in the meta-analysis

**Studies assessed for eligibility (n=142)**

**Records after duplicates removed (n=1982)**

**Records screened (n=1982)**

**Additional records identified through databases search (n=21)**

**Records identified through databases search (n=2687)**

**Records deleted based on title/abstract review (n=1840)**

**Records excluded (n=119)**

**Studies included in the final analysis (n=23)**

**Included**

**Eligibility**

**Screening**

**Screening**

**Table S3.** Main characteristics of included studies.

| **Reference** | **Type of DDS** | **Drug** | **Target/ligand (active/passive)** | **Species /strain / genotype** | **Age** | **Weight (Gram)** | **Cell type**  **(No. of cells)** | **Administration route** | **Follow up days** | **Outcome measures**  **(Tumor volume mm3)** |
| --- | --- | --- | --- | --- | --- | --- | --- | --- | --- | --- |
| Gao, 2013 ^23^ | Micelles | Dox | Passive | Balb/c female | ND | ND | CT26, 1×10⁶ | IV | 25 | Normal salts (NS) 2400mm3, Free Dox (Dox: 5mg/kg) 1000mm3, Dox/ SSMPEG-PCL (Dox: 5mg/kg) 600mm3 or SSMPEG-PCL (Control) 2200mm3. |
| Handali, 2018 ^24^ | Liposomes | 5-FU | FA (active) | Balb/c | ND | ND | CT26, 1×10⁶ | IP | 20 | Control 620mm3; 5FU-Free Drug 330mm3; Folate Liposome 5-FU 170mm3 |
| Handali, 2019* ^25^ | Liposomes | 5-FU | FA (active) | Balb/c male | ND | ND | CT26, 1×10⁶ | IP | 21 | Control 650mm3, 5-FU 350mm3, Folate Liposomal 5-FU 80mm3. |
| Handali, 2019** ^26^ | NP | 5-FU | Passive | Balb/c male | ND | ND | CT26, 1×10⁶ | IP | 22 | Control 620 mm3; 5-FU 280mm3; 5-FU loaded PHBV/PLGA 40mm3 |
| liu, 2015 ^27^ | NP | 5-FU | HA (active) | ND | ND | ND | Colo-205, 1×10⁶ | IV | 20 | Control 2400 mm3; 5-FU 1400 mm3; 5-FU/SNP 800mm3; 5-FU/HSNP 400mm3 |
| Jiang, 2017 ^28^ | NP | 5-FU | HA (active) | Balb/c | ND | ND | HT29, 1×10⁶ | ND | 21 | Control 1600mm3; 5-FU 1200mm3; FMSN 750mm3; HA/FMSN 400mm3 |
| Le, 2014 ^29^ | NP | 5-FU | FA (active) | Balb/c | 6 weeks | 20 g | HCT-8, 1×10⁶ | IP | 30 | Control-saline 1500mm3, blank NL/NP 1400mm3, free 5-FU 1100mm3, 5-FU/NP 800mm3 and FA/5-FU/NP 600mm3 |
| Li, 2008 ^30^ | NP | 5-FU | Passive | Balb/c | 6-8 weeks | 20–30 g | LoVo | IP | 21 | Control group (PBS) 430mm3, Empry PEG-PBLG 420mm3, 5-FU 120mm3, 5-FU/PEG-PBLG 100mm3 |
| Liang, 2014 (A) ^31^ | Liposomes | 5-FU | TGB6 monoclonal antibodies (active) | Balb/c female | ND | ND | HT-29, 1×10⁷ | IV | 21 | Control 1000mm3; blank imunoliposomes 970mm3; Free 5-FU 500mm3; Liposome+5-FU 300mm3; Targeted Imunoliposomes +5-FU 100mm3. |
| Liang, 2014 (B) ^31^ | Liposomes | 5-FU | TGB6 monoclonal antibodies (active) | Balb/c female | ND | ND | SW480b6 | IV | 21 | Control 900 mm3; blank imunoliposomes 850 mm3; Free 5-FU 650 mm3; Liposome+5-FU 400 mm3; Targeted Imunoliposomes +5-FU 190 mm3. |
| Liang, 2016 ^32^ | Micelles | GEM | Passive | ND | ND | ND | HT-29, 5×10⁶ | IV | 25 | Control 1600mm3; Blank Micelles 1500mm3; GEM 800mm3; GEM-PL 400mm3 |
| Moghimipour 2017 ^33^ | Liposomes | 5-FU | FA (active) | BALB/c male | ND | ND | CT26, 1×10⁶ | IP | 20 | Control 1200mm3;Free Drug 5-FU 200mm3; Folate-liposomal 5FU 100 mm3 |
| Ortiz, 2015 ^34^ | NP | 5-FU | Passive | C57BL/6 female | ND | 25–30 g | MC-38, 5×10⁵ | IV | 45 | Control (untreated mice) 9000mm3, PBCA 7000mm3, PBCA-5FU 2200mm3, 5-FU 4500mm3. Tumor volume PCL NPs Control 8500mm3, PCL 9500 mm3, PCL-5FU 1900mm3, 5-FU 4500mm3. |
| Pan, 2017 ^35^ | NP | 5-FU | Targeting peptide (RGD) (active) | Balb/c male | 4-6 weeks | ND | HCT116, 1×10⁶ | IV | 18 | Saline590 mm3 , MSN-P(OEGMA-co-RGD) 590mm3, free 5-FU 290mm3, 5-FU@MSN 200mm3, 5-FU@MSN-RGD 160mm3. |
| Sanati, 2020 ^36^ | Micelles | CPT | Apt (active) | Balb/c female | ND | ND | C26, 3×10⁵ | IV | 26 | Normal Saline 1400mm3; CPT 3mg/kg 900mm3; PLA/PEI-SUR-DEX 1000mm3; PLA/PEI-CPT-LUC-DEX 1100mm3; PLA/PEI-CPT-SUR-DEX 100mm3; PLA/PEI-CPT-SUR-DEX-APT 50mm3. |
| Shakeri-Zadeh, 2014 ^37^ | NP | 5-FU | Passive | Balb/c male | 6–8 weeks | 20–30 g | CT26, 2×10⁶ | IV | 21 | 5-FU 1800mm3, nanocapsules containing 3mg/kg 5-Fu, with a magnetic field 400mm3 |
| Song, 2018 (A) ^38^ | Micelles | SN-38-BOC | Passive | Balb/c | 6–8 weeks | ND | HCT116, 1×10⁷ | IV | 18 | Normal saline 250 mm3; Free Micele 1300; free SN-38-BOC (20 mg/kg) 900mm3 and SN-38-BOC micelles (20 mg/kg of SN-38-BOC) 50mm3 |
| Song, 2018 (B) ^38^ | Micelles | SN-38-BOC | Passive | Balb/c | 6–8 weeks | ND | CT26, 5×10⁵ | IV | 18 | Normal saline 1300m3; free micele 950mm3; free SN-38-BOC (20 mg/kg) 400mm3 and SN-38-BOC micelles (20 mg/kg of SN-38-BOC) 190mm3 |
| Wang, 2018 ^39^ | NP | 5-FU | Passive | Balb/c | 6–8 weeks | ND | SW480, 1×10⁶ | IV | 21 | Blank control 1000mm3; 5-FU 870mm3; ENCs 1100mm3; FNCs 300mm3; DNCs 1050mm3; DFNCs 250mm3 |
| Wu, 2020 ^40^ | NP | 5-FU | Epidermal growth factor (active) | Balb/c female | 6–8 weeks | 20–22 g | SW620, 3×10⁶ | IV | 20 | Control saline 790mm3, blank NPs (100 mg/kg) 790mm3, 5Fu (8 mg/kg) 640mm3, PLGA@5Fu (8mg/kg of 5Fu) 590mm3, PLGA@5Fu&PFC (8 mg/kg of 5Fu) 500mm3 or EGF-PLGA@5FU&PFC (8mg/kg of 5Fu) 390mm3. |
| Xu, 2015 ^41^ | Micelles | SN-38 | Passive | Nu/Nu strain/female | 5-6 weeks | 20 g | HT-29, 1×10⁷ | IV | 44 | Control 690mm3., Free drug 630mm3., NP+SN-38 560mm3. |
| Yang, 2017 ^42^ | Micelles | SN-38 | Passive | Nu/Nu strain/male | 6–8 weeks | ND | HT-29, 1×10⁶ | IV | 30 | Control (PBS) 1700mm3, CPT-11 850mm3, empty NPM+90J/cm2 500mm3, SN-NPM+90J/cm2 150mm3 |
| Yu, 2020 ^43^ | NP | DTX | Apt (active) | Balb/c female | 6–8 weeks | 18–22 g | CT26, 5×10⁵ | IP | 32 | Control 2150 ± 270.15 mm3; NPs-DTX 1236.61 ± 197.61 mm3; Apt-NPs- DTX 827.19 ± 140.71 mm 3 |
| Zhang, 2016 ^44^ | Liposomes | Dox | FA (active) | Balb/c nu/nu, female | 6–8 weeks | ND | CT26, 2×10⁶ | IV | 20 | Control 1200mm3 ; Free Dox 800mm3; FA-GDNVs 1100mm3; Dox-FA-GDNVs 450mm3 |
| Zhu, 2016 ^45^ | Micelles | 5-FU | Passive | ND | 8 weeks | ND | HCT116, 2×10⁶ | IV | 14 | Saline Control 110mm3; P85-90mm3; 5-Fu (1 mg/kg) 80mm3; 5-Fu (10 mg/kg) 70mm3 and 5-Fu (1 mg/kg)/P85-60mm3 |

***Abbreviations****:* Apt = Aptamer, CPT = Camptothecin, DEX = Dextran , DTX = Docetaxel, Dox = Doxorubicin, DNC = DNA-loaded nanocarrier, DFNC = DNA and 5-FU co-loaded nanocarrier, EGF = epidermal growth factor, ENC = empty nanocarrier, FA = Folic acid, 5-FU = 5-Fluorouracil, FNC = 5-FU-loaded nanocarrier, FMSN = Functionalized Mesoporous Silica NP, GDNV = Ginger-derived Nano-vectors, HA = Hyaluronic acid, HSNP = hyaluronic acid-conjugated silica , IP = Intraperitoneal, IV = Intravenous, MSN = Mesoporous silica NP, MPEG = Methoxy Poly(ethylene glycol), ND = Not described, NP = Nanoparticles, PHBV/PLGA = Poly(3-hydroxybutyrate-co-3-hydroxyvalerate)/Poly(lactic-co-glycolic acid), PEG = Polyethylene glycol, PBLG = Poly(γ-benzyl-L-glutamate), PBS = Phosphate-Buffered Saline, PBCA = Poly(butylcyanoacrylate), PCL = Poly(ε-caprolactone), P(OEGMA) = Poly(oligo(ethylene glycol) monomethyl ether methacrylate), PLA = Polylactic Acid, PEI = Polyethylenimine, P(CL-ran-TMC) = Poly(ε-caprolactone-ran-Trimethylene carbonate), PLGA = Poly(lactic-co-glycolic acid), PFC = perfluorocarbon, P85 = Poly (ethylene oxide) (PEO) blocks and hydrophobic poly (propylene oxide) (PPO) blocks, SNP = Silica NP, SN-NPM = SN-38-loaded photonic micelles, SUR = Survivin - shRNA (short hairpin RNA targeting survivin), SN-38 = 7-ethyl-10-hydroxycamptothecin, SN-38-BOC: SN-38 conjugated with a BOC (tert-butoxycarbonyl) protecting group, SSMPPEG-PCL = Stealth-Modified Methoxy Poly(ethylene glycol)-Poly(caprolactone), SSMPEG-PCL = Stealth-Modified Methoxy Poly(ethylene glycol)-Poly(caprolactone).

**Figure S2.** The efficacy of chemotherapy in inhibiting tumor growth compared to the control group. A) Tumor growth inhibition effects of chemotherapeutics in DDS and free form compared to the control group; B) Tumor growth inhibition effects of chemotherapeutics in a DDS vs. free chemotherapeutics as a control group.


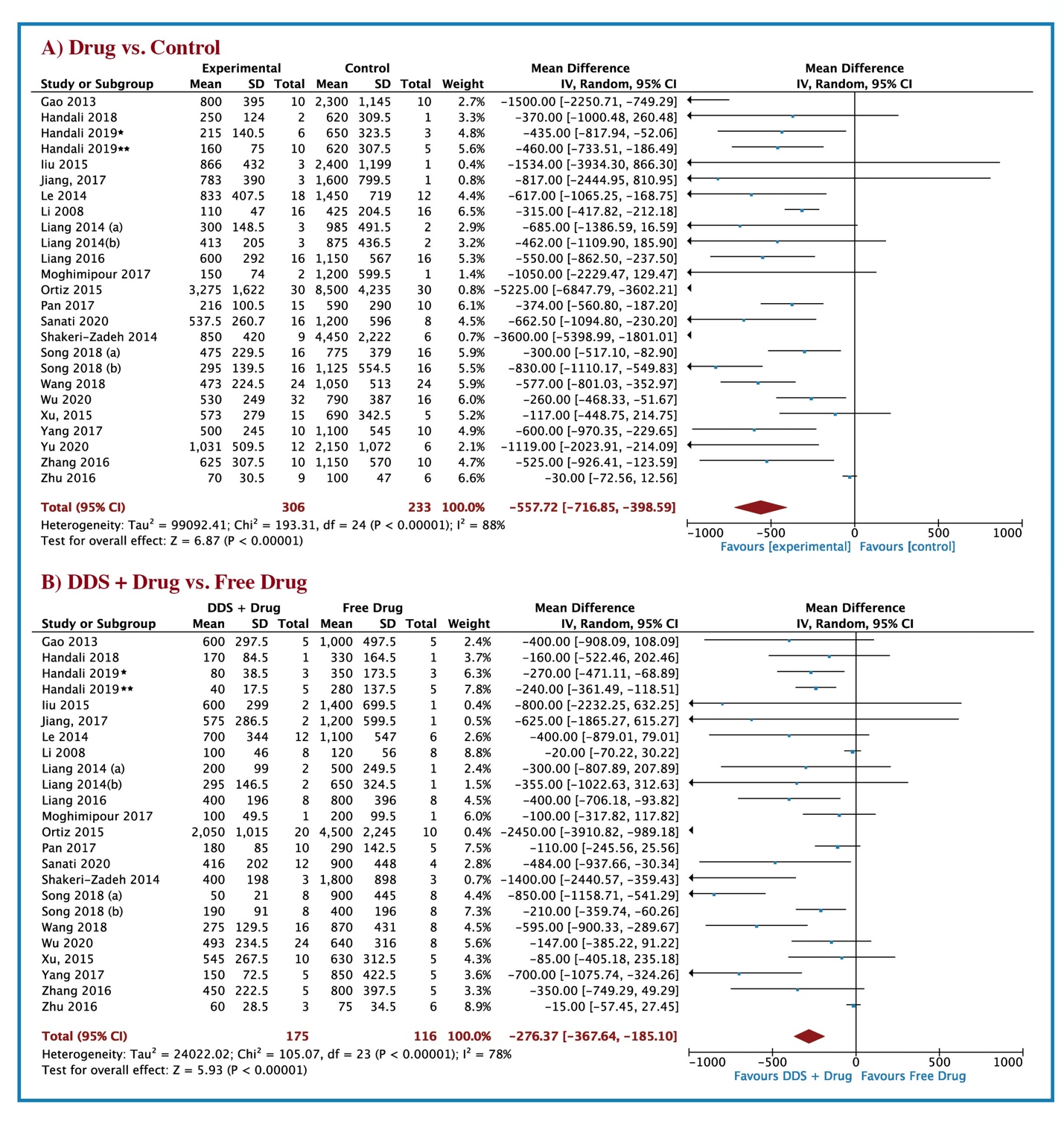


*Legend: DDS: Drug delivery system*

**Figure S3.** Comparison of the efficacy of targeted versus non-targeted DDS in combination with drug therapy on tumor growth inhibition. A) Tumor growth inhibition effects of chemotherapeutics in targeted DDS compared to chemotherapeutics in non-targeted DDS; B) Tumor growth inhibition effects of chemotherapeutics in targeted DDS or non-targeted DDS compared free chemotherapeutics.


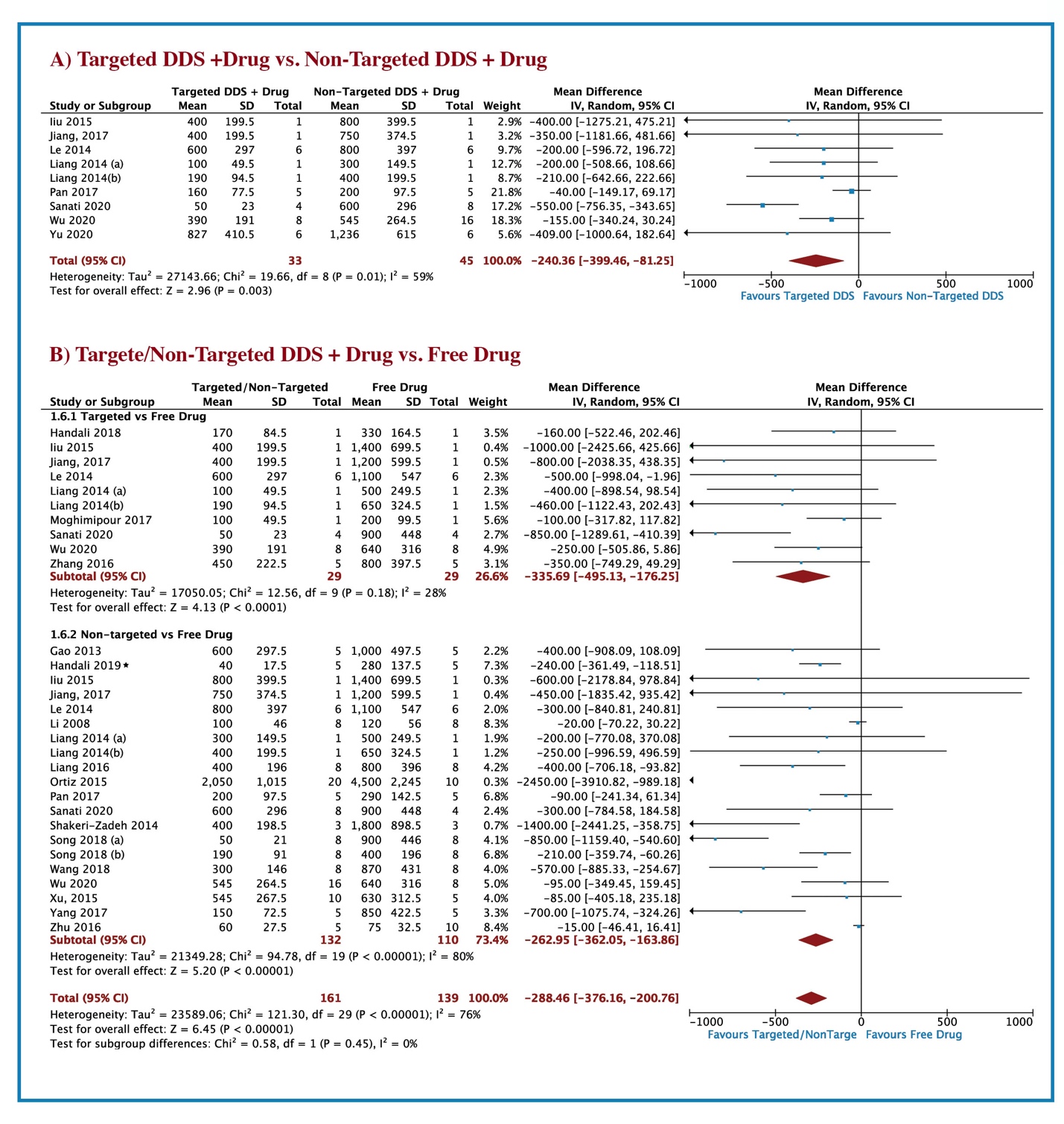


*Legend: DDS: Drug delivery system*

**Figure S4.** The efficacy of chemotherapeutics in various DDS types compared to free chemotherapeutics on tumor growth inhibition in subgroup analysis: Micelles, Liposomes, and Nanoparticles.


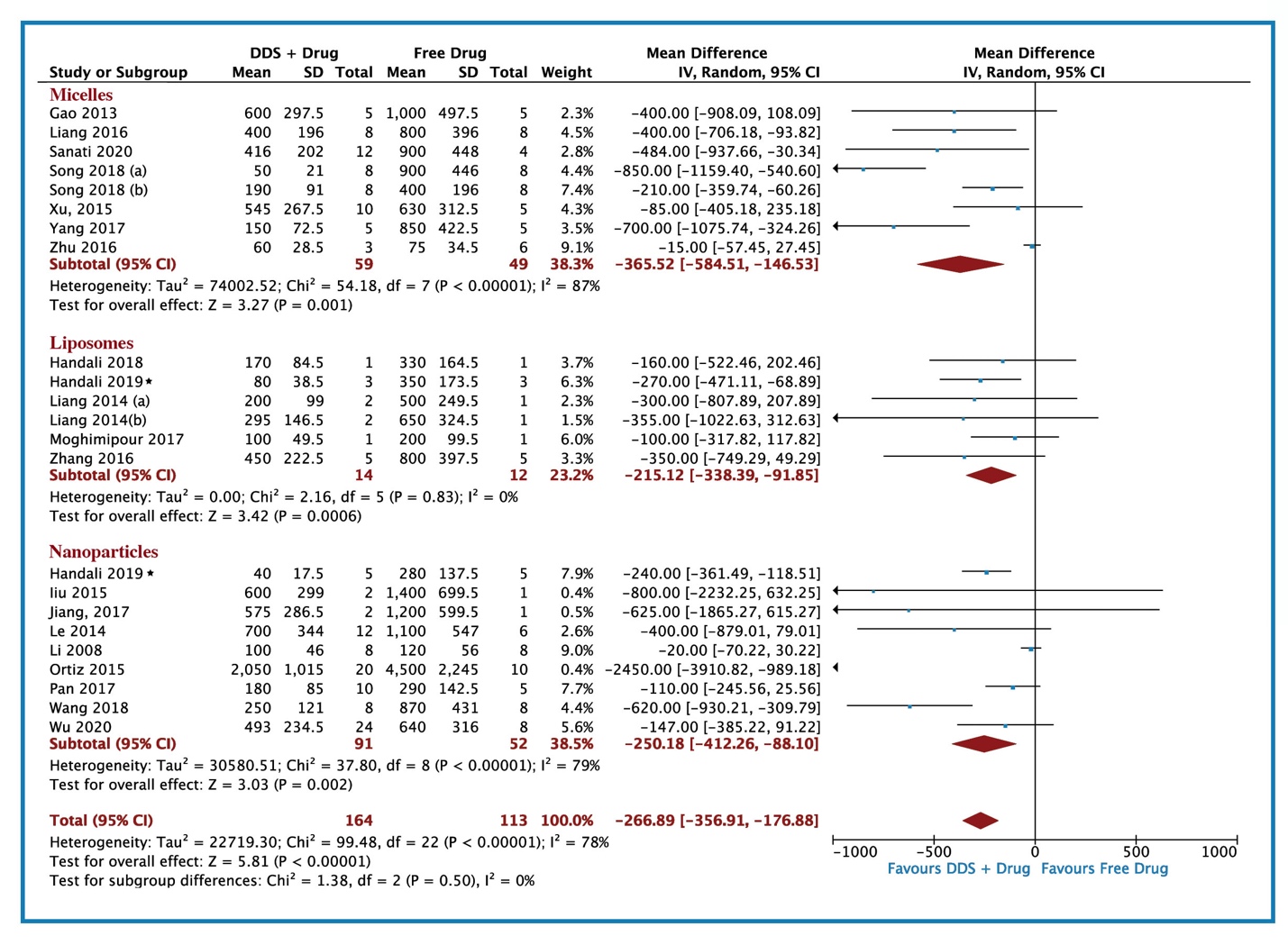


*Legend: DDS: Drug delivery system*

**Figure S5.** Tumor growth inhibition effects of different chemotherapeutics in a DDS compared to free chemotherapeutics in subgroup analysis: Fluorouracil, Doxorubicin, SN-38.

*
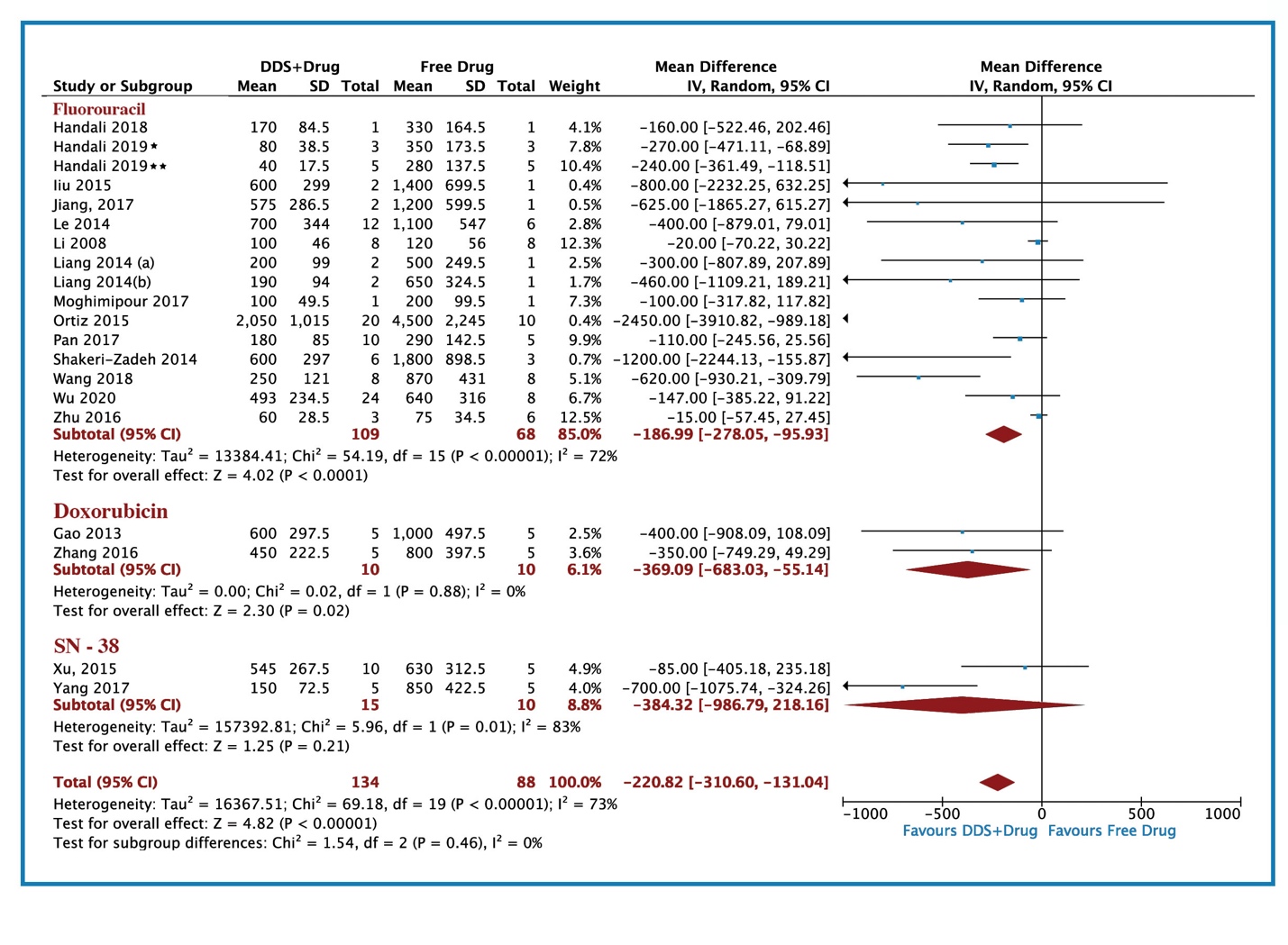
*

*Legend: DDS: Drug delivery system*

**Figure S6.** The efficacy of chemotherapeutics in DDS compared to free chemotherapeutics on tumor growth inhibition in subgroup analysis: Intravenous, Intraperitoneal

*
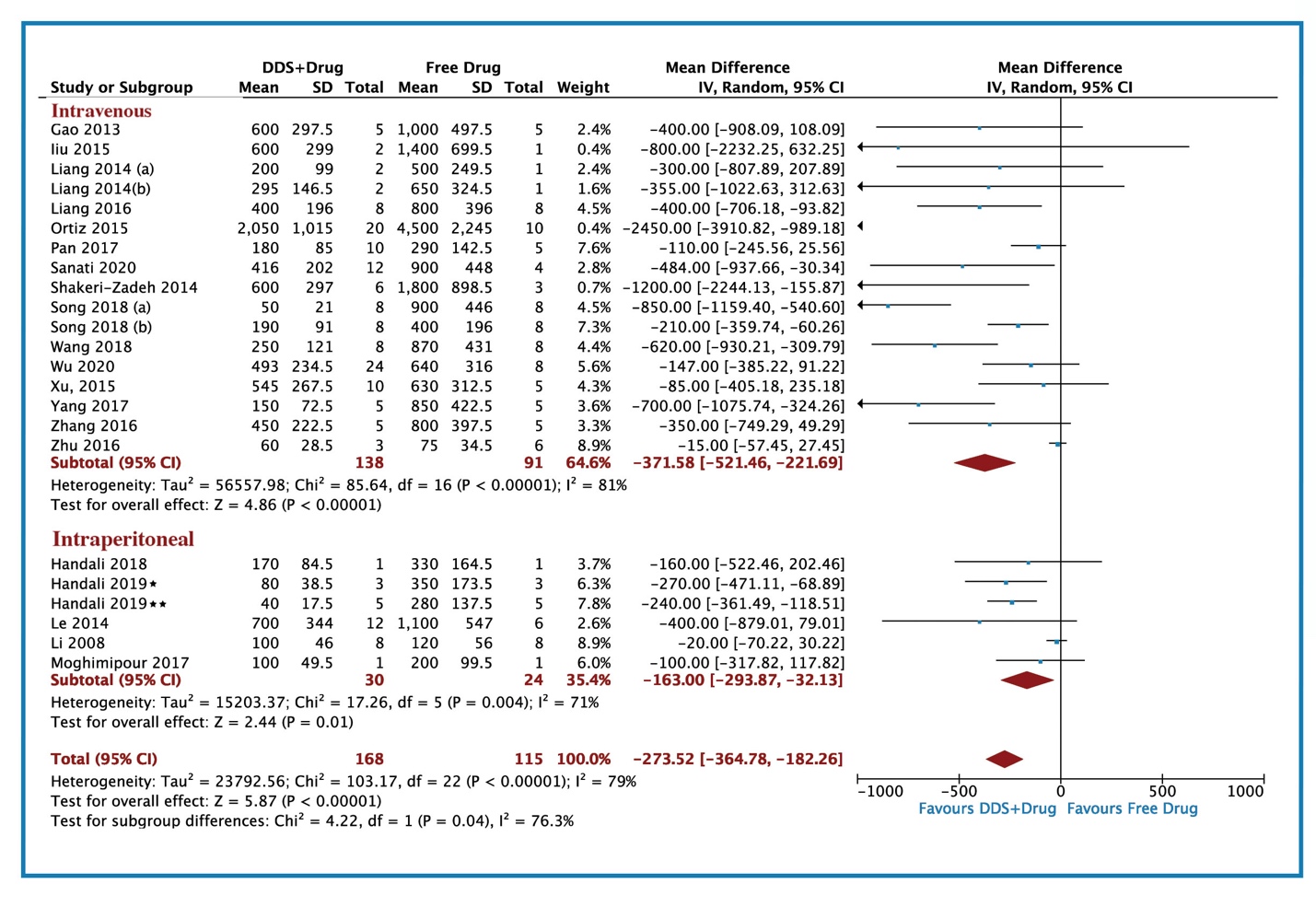
*

*Legend: DDS: Drug delivery system*

**Figure S7.** The efficacy of chemotherapeutics in targeted DDS compared to free chemotherapeutics on tumor growth inhibition in subgroup analysis: Folic acid; Aptamer; Hyaluronic acid

*
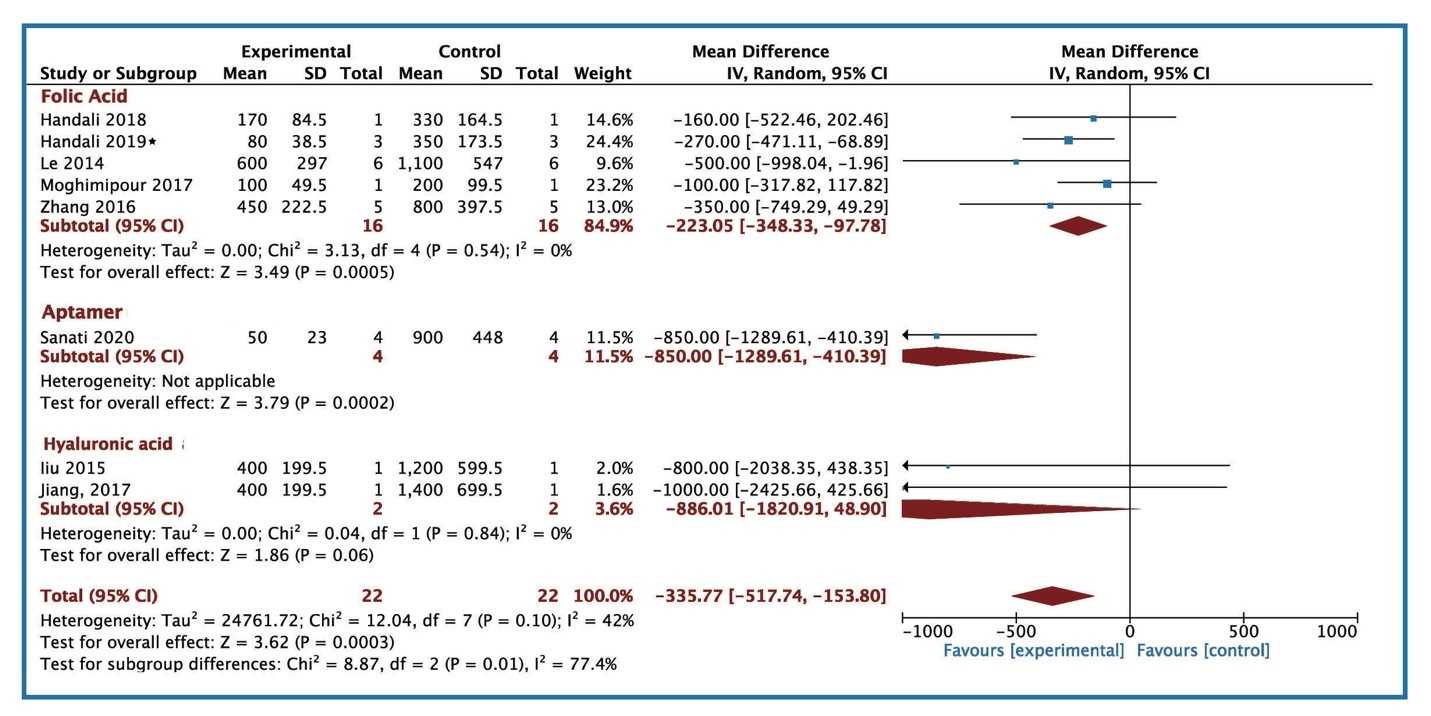
*

*Legend: DDS: Drug delivery system*

**Table S4.** Assessment of risk of bias in the included studies using SYRCLE’s RoB tool for animal studies.

| **References** | **Is it mentioned that the experiment was randomized?** | **Is it mentioned that the experiment was blinded (level unknown)?** | **Is a power/sample size calculation shown?** | **Was the allocation sequence adequately generated and applied?** | **Was the allocation adequately concealed?** | **Were the caregivers/ and or investigators during the course of the experiment blinded from knowledge of which intervention each animal received?** | **Was the outcome assessor blinded?** | **Are the animals randomly housed during the experiment?** | **Were animals selected at random for the outcome assessment?** | **Were the groups similar at baseline or was adjusted for confounders in the analysis?** | **Were incomplete outcome data adequately addressed?** | **Was the study apparently free of other problems that could pose a high risk of bias?** |  |
| --- | --- | --- | --- | --- | --- | --- | --- | --- | --- | --- | --- | --- | --- |
|  |  |  |  |  |  |  |  |  |  |  |  |  |  |
|  |  |  |  |  |  |  |  |  |  |  |  |  |  |
|  |  |  |  |  |  |  |  |  |  |  |  |  |  |
| G+B10:Q34ao, 2013 ^23^ | Y | N | N | N | U | U | U | U | U | Y | U | U |  |
| Handali, 2018 ^24^ | N | N | N | U | U | U | U | U | U | Y | U | N |  |
| Handali, 2019 (A) ^25^ | Y | N | N | U | U | U | U | U | U | Y | U | U |  |
| **Handali, 2019 (B) ^26^** | Y | N | N | U | U | U | U | U | U | Y | U | U |  |
| liu, 2015 ^27^ | Y | N | N | U | U | U | U | U | U | U | U | U |  |
| Jiang, 2017 ^28^ | N | N | N | N | U | U | U | U | U | U | U | N |  |
| Le, 2014 ^29^ | Y | N | N | N | U | U | U | U | U | Y | N | U |  |
| Li, 2008 ^30^ | N | N | N | N | U | U | U | U | U | Y | Y | N |  |
| Liang, 2014 (B) ^31^ | Y | N | N | U | U | U | U | U | U | Y | N | U |  |
| Liang, 2016 ^32^ | N | N | N | U | U | U | U | U | U | N | U | U |  |
| Moghimipour, 2017 ^33^ | Y | N | N | U | U | U | U | U | U | U | N | U |  |
| Ortiz, 2015 ^34^ | Y | N | N | U | U | U | U | U | U | U | Y | Y |  |
| Pan, 2017 ^35^ | Y | N | N | U | U | U | U | U | U | N | N | U |  |
| Sanati, 2020 ^36^ | Y | N | N | U | U | U | U | U | U | U | Y | Y |  |
| Shakeri-Zadeh, 2014 ^37^ | Y | N | N | U | U | U | U | U | U | U | U | U |  |

| Song, 2018 (B) ^38^ | Y | N | N | U | U | U | U | U | U | Y | Y | Y |
| --- | --- | --- | --- | --- | --- | --- | --- | --- | --- | --- | --- | --- |
| Wang, 2018 ^39^ | N | N | N | N | U | U | U | U | U | Y | Y | U |
| Wu, 2020 ^40^ | Y | N | N | U | U | U | U | U | U | U | U | U |
| Xu, 2015 ^41^ | Y | N | N | U | U | U | U | U | U | U | U | U |
| Yang, 2017 ^42^ | Y | N | N | U | U | U | U | U | U | N | U | U |
| Yu, 2020 ^43^ | Y | N | N | U | U | U | U | U | U | Y | U | Y |
| Zhang, 2016 ^44^ | Y | N | N | U | U | U | U | U | U | Y | U | Y |
| Zhu, 2016 ^45^ | Y | N | N | U | U | U | U | U | U | Y | U | Y |
| **Yes (Low)** | 78% | 0% | 0% | 0% | 0% | 0% | 0% | 0% | 0% | 52% | 21% | 26% |
| **Unclear** | 0% | 0% | 0% | 79% | 100% | 100% | 100% | 100% | 100% | 35% | 61% | 61% |
| **No (High)** | 22% | 100% | 100% | 21% | 0% | 0% | 0% | 0% | 0% | 13% | 18% | 13% |

| **Yes (L)** | *Low risk of bias* |
| --- | --- |
| **No (H)** | *High risk of bias* |
| **Unclear (U)** | *If insufficient details have been reported to assess* |
|  | *the risk of bias properly.* |
